# Targeting transforming growth factor-β1 by methylseleninic acid/seleno-L-methionine in clear cell renal cell carcinoma: Mechanisms and therapeutic potential

**DOI:** 10.1016/j.ctarc.2025.100864

**Published:** 2025-01-09

**Authors:** Aseel O. Rataan, Yan Xu, Sean M. Geary, Yousef Zakharia, Eman S. Kamel, Youcef M. Rustum, Aliasger K. Salem

**Affiliations:** a Department of Pharmaceutical Sciences and Experimental Therapeutics, College of Pharmacy, University of Iowa, Iowa City, IA 52242, USA; b Department of Clinical Pharmacy and Pharmacy Practice, Faculty of Pharmacy, Yarmouk University, Irbid, Jordan; c Department of Internal Medicine, Division of Hematology and Oncology, University of Iowa, Iowa City, IA 52242, USA; d Holden Comprehensive Cancer Center, University of Iowa, Iowa City, IA 52242, USA; e Department of Internal Medicine, Carver College of Medicine, University of Iowa, Iowa City, IA 52242, USA; f Roswell Park Comprehensive Cancer Center, Department of Pharmacology & Therapeutics, Buffalo, NY 14203, USA

## Abstract

Clear cell renal cell carcinoma (ccRCC) poses a significant global health challenge as its incidence continues to rise, resulting in a substantial annual mortality rate. Major clinical challenges to current ccRCC treatments include high drug-resistance rates as well as dose-limiting adverse events; underlining the need to identify additional ‘druggable’ targets. TGF-β1, VEGF, and PD-L1 are potential therapeutic targets in ccRCC. This study analyzed their expression in human ccRCC cell lines and patient tumor biopsies. Data obtained from western blotting demonstrated higher levels of TGF-β1 and PD-L1 and lower levels of VEGF in sarcomatoid ccRCC cell lines compared to non-sarcomatoid ccRCC cell lines. In patient samples, TGF-β1 was significantly upregulated in both non-sarcomatoid and sarcomatoid ccRCC tumors. It was demonstrated through two assays (cellular thermal shift assay and a size exclusion assay) that methylseleninic acid (MSA) binds specifically and directly to TGF-β1. MSA treatment significantly downregulated TGF-β1, PD-L1, and VEGF in a dose- and time-dependent manner in both non-sarcomatoid and sarcomatoid ccRCC cell lines. Seleno-L-methionine (SLM) treatment in a nude mouse xenograft model showed a significant tumor growth inhibition and TGF-β1 downregulation at non-toxic doses. These findings suggest that selenium-mediated downregulation of TGF-β1, PD-L1, and VEGF could be a viable therapeutic strategy for ccRCC.

## 1. Introduction

Renal cell carcinoma (RCC) is the second most common malignancy arising from the urinary system in the United States. According to the American Cancer Society’s estimates in 2023, there will be ~81,800 new cases of RCC, and it is expected to cause approximately 14,890 deaths [1]. Clear cell RCC (ccRCC) accounts for 70–85 % of RCC cases and arises from the epithelial layer of the proximal convoluted tubule of the kidney [2]. It is characterized by: loss of the von-Hippel Lindau (VHL) tumor suppressor gene in approximately 90 % of cases [3]; cytoplasmic lipid accumulation [4]; high vascularity [5]; and high levels of expression of TGF-β1 [6]. ccRCC is considered one of the most lethal cancers with high resistance rates to conventional chemotherapy and radiotherapy [7]. Tyrosine kinase inhibitors and immune checkpoint inhibitors (ICI) (e.g., anti-programmed death-ligand 1/programmed death-1 (anti-PD-L1/PD-1)) are among the most commonly used therapies (alone and in combination) for ccRCC particularly in advanced metastatic patients [8–10]. Approximately 5–15 % of ccRCC tumors are sarcomatoid and characterized by: poor prognosis; low response to standard of care therapies; express mutated TP53; and express upregulated levels of PD-L1 [11]. Drug resistance, *de novo* and acquired, and serious adverse events continue to impose major clinical impediments to current therapeutic approaches [12] and provide the motive to identify and evaluate alternative ‘druggable’ targets aimed at enhancing the efficacy and mitigating resistance of standard therapies in patients with advanced ccRCC.

TGF-β is a multifunctional cytokine that plays an important role in cellular growth and development, as well as host immunity and inflammation. There are three main mammalian isoforms for TGF-β (TGF-β1, TGF-β2 and TGF-β3) [13]. In cancer, TGF-β1 plays contrasting roles; inhibiting cellular transformation and tumor progression in the early stages of cancer, while stimulating tumor growth in the later stages of the disease by either promoting epithelial-to-mesenchymal transition (EMT), inducing angiogenesis, or triggering immunosuppression [14]. Bostrom et al. demonstrated that inhibition of TGF-β1 attenuates the invasive capacity of ccRCC cells [15]. Another distinguishing feature of ccRCC is that it is regarded as a silent disease, with most cases being discovered by chance and late enough to have spread to distant organs. Approximately 30 % of patients with ccRCC initially present with advanced disease [16], while approximately 30 % of patients that initially present with localized tumors will progress to advanced disease after nephrectomy [6]. This poses a huge challenge in finding treatments with high efficacy, low off-target toxicity, and high response rates.

Methylseleninic acid (MSA) is a reactive organoselenium compound that is used in vitro as a substitute for seleno-l-methionine (SLM) which is mostly utilized for in vivo studies and clinical trials. SLM is not active in vitro and needs the action of the β-lyase enzyme (not present in most cancer cell lines) to become active [17]. Selenium compounds have been shown to have an impact on ccRCC treatments by stabilizing tumor vasculature, which improves the delivery of chemotherapeutic agents to the tumor and increases the therapeutic efficacy of several drugs in ccRCC xenografts, including targeted therapies [18–20]. Additionally, several studies demonstrated that selenium compounds could sensitize tumor cells to various chemotherapies by inhibiting hypoxia-inducible factors (HIFs) expression which in turn inhibit the expression of PD-L1 and VEGF [21–25]. There are limited studies exploring the role of selenium compounds in affecting TGF-β1 expression. A previous study has shown that selenium selenite can downregulate TGF-β1 expression in a prostate cancer cell line [26]. However, the effect of selenium compounds on TGF-β1 in ccRCC is yet to be investigated either phenomenologically or mechanistically and the present study aimed to investigate whether TGF-β1, PD-L1, and VEGF are important therapeutic targets in ccRCC, and, if so, to also determine whether TGF-β1 is a specific, direct selenium target. This study presents data that provides an understanding of the mechanism of TGF-β1 inhibition by selenium (MSA in vitro and SLM in vivo) and suggests that selenium compounds can be an additional option for the treatment of patients with advanced ccRCC and other cancers expressing selenium targets.

## 2. Materials and methods

### 2.1. Cell culture and drugs

#### 2.1.1. Cell culture

The ccRCC cell lines, 786-O and Caki-1 were purchased from American Type Culture Collection (ATCC) (Manassas, VA). The cell line, 786-O was maintained in RPMI-1640 (Gibco, Invitrogen, Waltham, MA) containing 10 % (v/v) fetal bovine serum (FBS) (Atlanta Biologicals, Lawrenceville, GA) and 1 % penicillin-streptomycin (100 U/mL; Gibco), while Caki-1 was maintained in McCoy’s 5A (ATCC) containing 10 % (v/v) FBS and 1 % penicillin-streptomycin (100 U/mL). RCC4, RCC4/VHL and HK-2 were a kind gift from Dr. Steven Eliason’s laboratory/Department of Anatomy and Cell Biology at the University of Iowa whereas the RC2 cell line was a generous gift from Dr. Christopher Stipp’s laboratory/ Department of Biology at the University of Iowa (Iowa City, IA). RCC4, RCC4/VHL, HK-2 and RC2 were cultured in Dulbecco’s modified Eagle’s medium (DMEM) (Gibco) supplemented with 10 % (v/v) FBS, 30 U/ml penicillin-streptomycin and 1 % (v/v) geneticin (G418) (10 mg/ml; Gibco) was added for RCC4 and RCC4/VHL cell lines to main stable VHL expression (Gibco). Recently derived sarcomatoid RCC cell lines from Mayo Clinic, RCJ41T1, RCJ41M, RCJ41T2 were kindly provided by Dr. Thai H. Ho (Phoenix, AZ). The three sarcomatoid cell lines were grown in DMEM (Gibco) supplemented with 10 % (v/v) FBS and 1 % penicillin-streptomycin (100 U/ml; Gibco). Renal proximal tubules epithelial cell line (RPTEC) was obtained from ATCC and grown in renal epithelial cell basal medium supplemented with renal epithelial cell growth kit which contains 0.5 % (v/v) FBS, 10 nM triiodothyronine, 10 ng/mL rh EGF, 100 ng/mL hydrocortisone hemisuccinate, 5 μg/mL rh insulin, 1 μM epinephrine, 2.4 mM L-alanyl-L-glutamine and 5 μg/ml transferrin (ATCC). Whereas UOK127 cell line was obtained from Dr. Marston Linehan from National Cancer Institute. UOK127 was maintained in DMEM media with 10 % (v/v) FBS and 100 U/ml penicillin-streptomycin. All cell lines were mycoplasma-free as determined by a MycoAlert mycoplasma detection kit (Lonza, Basel, Switzerland). All cells were grown and maintained at 37 °C and 5 % CO_2_ flow in a humidified incubator (Sanyo Scientific Autoflow, IR direct heat CO_2_ incubator). Cells were not allowed to achieve confluency in any of the experiments and were kept in log phase growth throughout.

#### 2.1.2. Drugs

MSA and SLM were purchased from Sigma-Aldrich (St. Louis, MO). MSA is a white crystalline powder, and it was prepared by dissolving the powder in phosphate-buffered saline (PBS) for in vitro use. Whereas for in vivo use, SLM (a white powder) was dissolved in deionized water and freshly prepared at 2 mg/ml and doses were given by oral gavage based on mouse weight.

### 2.2. Animals

Female athymic NCI nude mice (nu/nu, body weight, 20–25 g), 6–8 weeks of age, were obtained from Charles River Laboratories (Wilmington, MA) and housed at 5 mice/cage with water and food ad libitum according to Institutional Animal Care and Use Committee approval (protocol # 0112351). Nude mice were used to avoid xenograft rejection of the 786-O human ccRCC cell line used here. Nude mice were subcutaneously challenged with 5 × 10^6^ 786-O cells. The mice were randomized into two groups and were treated with either vehicle (deionized water) or 10 mg/kg SLM. Treatment was administered by oral gavage daily for 45 days. Treatment with either the vehicle or 10 mg/kg SLM began on the same day as the tumor challenge. This experiment was done once. Body weights were monitored throughout the experiment, and tumor axes were measured twice weekly by digital caliper. Three axes of the tumor (L, longest axis in mm; W, shortest axis in mm; and H, height axis in mm) were measured and the tumor volume (mm^3^) was calculated based on the following formula:

$$\text{Tumor}\;\text{volume}\left({\text{mm}}^{3}\right)=\left(\pi /6\right)\times \text{L}\times \text{W}\times \text{H}$$

Mice were euthanized when the longest diameter reached 20 mm, tumor height reached 10 mm, or its body weight dropped by 20 % from the baseline weight. To determine the acute toxicity and maximum tolerated dose of SLM, female NCI athymic nude mice were treated with 6,8,10,12,16,20 and 29 mg/kg of SLM for 3 days and they were monitored for body weight reduction and lethality for up to 4 days after discontinuation of SLM. The experiment was conducted once with *n* = 5 mice per group.

### 2.3. Tissue extraction and protein quantification

Total protein was extracted from paired normal and tumor resection specimens from 47 patients (enrolled in a clinical trial at the UIHC). Tissues were weighed and cut into ~ 10 mg pieces. For each 50 mg of tissue, 200 μl of tissue extraction reagent I (ThermoFisher Scientific, Waltham, MA) premixed with 4 μl protease inhibitor cocktail (Santacruz Biotechnology, Dallas, TX), 2 μl sodium orthovanadate solution (Santacruz Biotechnology) and 2 μl phenylmethylsulfonyl fluoride solution (Santacruz Biotechnology) were added. The mixture was then homogenized for two minutes using the Fisher brand Bead Mill 4 (Fisher scientific) at speed 5. All the previous steps were performed at 4 °C. The mixture was then moved to a 1.5 ml Eppendorf tube and centrifuged for 10 min at 4 °C at 14,000 g. The supernatant was collected, and total protein was quantified using a Micro Pierce^™^ BCA Protein Assay Kit (ThermoFisher Scientific). Samples (30 μg total protein) were mixed with 4X Laemmli sample buffer (Bio-Rad Laboratories, Hercules, CA) premixed with β-mercaptoethanol (VWR International Incorporated, Radnor, PA). The samples were then incubated at 95 °C for 5 min, loaded into the wells of precast 4–20 % Mini-Protein gels (Bio-Rad Laboratories) and an electric current was applied using a power supply (110 V, 400 mA) for 70 min. Proteins in the gel were then transferred to 0.2 μm nitrocellulose membrane (Bio-Rad Laboratories, Hercules, CA) using a Bio-Rad Trans-Blot Turbo Transfer System. The membrane was then exposed to 5 % (w/v) bovine serum albumin (BSA) (Research Products International, Mount Prospect, IL) in washing buffer overnight at 4 °C and then washed with tris-buffered saline with 0.1 % tween-20 (TBST buffer) thrice each for 10 min/wash. Primary antibodies against PD-L1 (Cell Signaling Technology, Danvers, MA), TGF-β1 (abcam), VEGF (Millipore Sigma) and β-actin (Cell Signaling Technology) were diluted (1:1000) in 0.5 % (v/v) BSA in TBST buffer and incubated with the blots overnight at 4 °C and then washed with TBST buffer thrice for 10 min. The membrane was then probed with corresponding horseradish peroxidase-conjugated goat anti-rabbit/anti-mouse IgG (1:10,000 v/v) (Cell Signaling Technology) for 1 h at room temperature. The signal was visualized by chemiluminescence using Clarity^™^ Western ECL substrate (Luminol:H_2_O_2_ in 1:1 ratio) (Bio-Rad Laboratories) for 5 min and iBright FL1000 imaging system (ThermoFisher Scientific).

### 2.4. Cell lysis and protein quantification

After being treated with different MSA concentrations for various time points, 2 × 10^6^ cells from different cell lines were lyzed using 100 μl of Radioimmunoprecipitation assay buffer (RIPA buffer) premixed with protease inhibitor cocktail, sodium orthovanadate and phenylmethylsulfonyl fluoride solution (Santacruz Biotechnology, Dallas, TX). The mixture of the cells and the RIPA buffer was kept for 10 min on ice before being centrifuged at 4 °C at 14,000 g. Then the supernatant was collected, and the total protein was quantified using a Micro Pierce^™^ BCA Protein Assay Kit. Samples were prepared and analyzed as in Section 2.3.

### 2.5. Analyzing the interaction between MSA and TGF-β1

Two experiments were designed to determine if TGF-β1 and MSA directly interact. The first experiment involved mixing MSA in a 1:2 molar ratio with either recombinant TGF-β1, BSA or FBS in 1.5 ml Eppendorf tubes for 2 h at 4 °C using a tube rotator. The mixture was then passed through a 3 kDa pore filter to exclude TGF-β1 and selenium bound to TGF-β1 (MW of TGF-β1 is 25 KDa). After collecting the filtrate, the selenium level in each tube was determined by inductively coupled plasma – mass spectrophotometer (ThermoFisher Scientific, Waltham, MA) and compared with MSA alone samples. For the second experiment, involving the cellular thermal shift assay [27], 786-O cells were seeded in four 10 cm^2^ cell culture dishes with fresh media (RPMI + 10 % (v/v) FBS + 1 % (100 U/ml) penicillin-streptomycin) one day before the experiment. On the day of the experiment, the media of four 80–90 % confluent 10 cm^2^ dishes was replaced with 10 ml of fresh media and different treatments including PBS, 5 μM MSA, and 50 μM metformin. Metformin was utilized as a positive control in our experiments because it has been demonstrated in previous research to be a novel suppressor of TGF-β1 [28]. Cells were incubated with treatments for two hours at 37 °C and 5 % CO_2_. Subsequently, cells were trypsinized and collected into 15 ml tubes. The tubes were centrifuged at 300 g for 5 mins at 25 °C. Cell pellets were then resuspended in 10 ml PBS and counted. Cells were then centrifuged again, and the cell pellet was resuspended in ice-cold PBS (+ protease inhibitor) to prepare 12 × 10^6^ cells/ml suspension. The cell suspension was distributed into 12 different 0.2 ml PCR tubes with 50 μl of cell suspension in each tube (~600 000 cells per tube). PCR-tubes were then heated at their designated temperature for 3 min in a thermal cycler then, immediately after heating, the tubes were removed and incubated at room temperature for 3 min. The samples were then immediately snap-frozen in liquid nitrogen. The cells were freeze-thawed three times using liquid nitrogen and a thermal cycler set at 25 °C to ensure a uniform temperature between tubes. The tubes were vortexed briefly after each thawing step. The resulting cell lysates were kept on ice after the last thawing step. The tubes containing the cell lysate were briefly vortexed and centrifuged at 20,000 g for 20 min at 4 °C to pellet cell debris together with precipitated and aggregated proteins. The tubes were carefully removed from the centrifuge to avoid disturbing the pellets and the samples were kept on ice. Finally, the supernatants were analyzed using a western blotting technique as described in Section 2.3.

### 2.6. Selenium dose-dependent and time-dependent treatments of ccRCC cell lines and TGF-β1 expression recovery

For the dose-dependent experiments, RCJ41T1, RCJ41T2, RCJ41M and 786-O were seeded in 1 × 10^5^ cells/well in 6-well plates one day before the experiment. Cells were then treated with 0.1, 1 and 2.5 μM MSA for 24 h. After that, cells were lyzed, and protein content was analyzed as described in Section 2.3. Similarly, for the time-dependent experiment, cells were seeded into 6-well plates at 1 × 10^5^ cell/well and after 24 h, cells were treated with 2.5 μM MSA for 2, 4, 8, 16 and 24 h. After that, cells were lyzed, and the protein content was analyzed as described in Section 2.3.

To measure the recovery time, 1 × 10^5^ cells were seeded into 6-well plates, allowed to grow for 24 h, and then treated with 2.5 μM MSA for 24 h. After 24 h, cells were washed three times with PBS to remove residual MSA and fresh media was added. Samples were collected after 2, 4, 6, 8 and 24 h. Protein samples were analyzed as described previously in Section 2.3.

### 2.7. Generating a TGF-β1 knock-out in the 786-O cell line using CRISPR/Cas9

To generate the 786-O cell line with TGF-β1 knocked out (TGF-β1 KO), a lentivirus expressing eGFP (HIVmPKG-eGFP) (Viral Vector Core, Iowa City, IA) with a titer of 1.7 × 10^9^ transducing units/milliliter (TU/ml) was initially used to estimate the multiplicity of infection (MOI). A working concentration of 1 × 10^6^ TU/ml of the virus was prepared by adding 5.9 μl of the concentrated virus to 1 ml of RPMI media. In a 24-well plate, 1 × 10^5^ cells/well of 786-O cells were seeded in antibiotic-free RPMI medium containing 10 % (v/v) FBS (complete medium). The cells were allowed to grow for 24 h. After 24 h of incubation, the medium was removed and a mixture of 4 μg/ml polybrene (Sigma-Aldrich), and complete medium was added to the cells. Various amounts of HIVmPKG-eGFP lentivirus were added to the cells according to the following formula:

$$\text{Amount}\;\text{of}\;\text{virus}\;\text{to}\;\text{be}\;\text{added}\left(\mu l\right)=\text{MOI}\times \left(\#\text{of}\;\text{cells}\right)÷\text{titer}\;\text{of}\;\text{the}\;\text{virus}$$

The cells were incubated with the virus for 4 h. Then the medium was aspirated, and fresh complete medium was added. After 48 h, the cells were collected by trypsinizing the cells and passing them through a 100 μm cell strainer (Corning Inc, NY). The transduction efficiency was then determined using a flow cytometer (FACScan, Becton Dickinson, Franklin Lakes, NJ).

After determining the optimal MOI to be 200, 786-O cells were seeded in complete medium at 1 × 10^4^ cells/well in a 6 well-plate. After 24 h, 4 μg/ml of polybrene in complete medium was added. Lentivirus (200 MOI) with CRISPR/Cas9 designed to knock out TGF-β1 (gRNA sequence: TGTACAACAGCACCCGCGAC) (Viral Vector Core) and harboring puromycin resistance was also added for 4 h. Then, the medium was replaced with fresh complete medium. After 48 h, the medium was removed and media containing 2 μg/ml puromycin was added to select cells with TGF-β1 knocked out and possessing puromycin resistance. Medium was changed every 3 days. Protein samples were collected after 7 days of selection to check for the expression of TGF-β1 via western blotting (as described in Section 2.3).

### 2.8. Statistical analysis

All the in vitro experiments were repeated at least three times. Data are expressed as mean ± SD. Statistical analysis was performed using GraphPad prism software for Windows version 9.0.0 (GraphPad Software, Inc., San Diego, CA). One-way analysis of variance (ANOVA) followed by Tukey post hoc test was used to compare between 3 or more groups and a paired- *t*-test or Wilcoxon signed-rank test were used to compare between two groups. Differences were considered significant at *P* < 0.05.

For western blot analysis, image files were processed and analyzed using Image J software (1.53c, Wayne Rasband, National Institutes of Health, USA). Briefly, the image was cropped to include only the bands of interest and the immediate surrounding areas of the blot. The actual intensities of the bands were calculated, and the data was presented as the intensities of the protein of interest relative to the housekeeping protein.

## 3. Results

### 3.1. Assessing TGF-β1, PD-L1 and VEGF as potential therapeutic targets through relative protein expression in normal and RCC tissues and cell lines

**Fig. 1.**
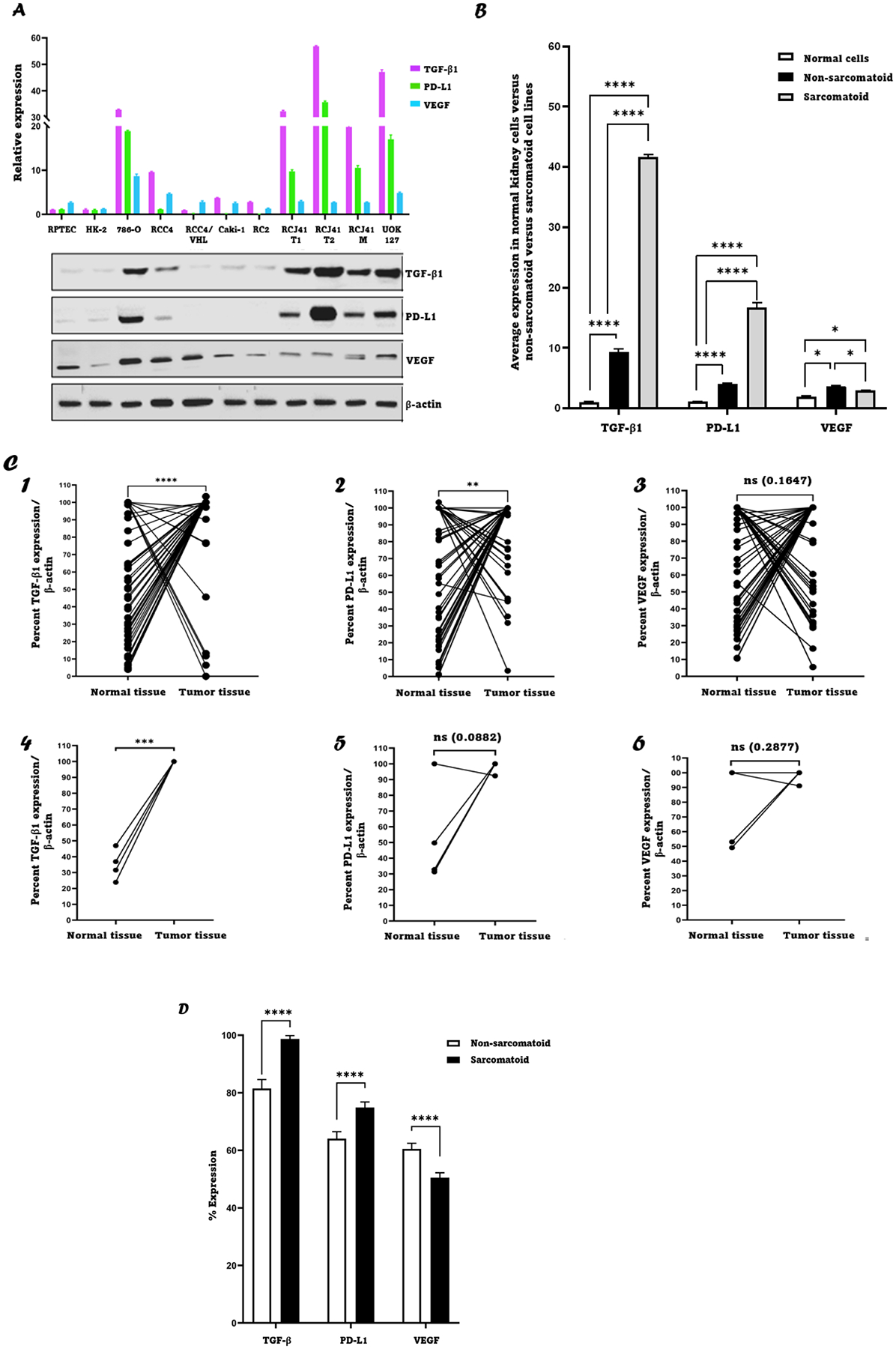
TGF-β1, PD-L1, and VEGF expression in tumor tissues and ccRCC cell lines. **A:** (*Top*) Relative expression of TGF-β1, PD-L1, and VEGF by indicated ccRCC cell lines and normal kidney cell lines (RPTEC and HK-2) as deciphered from the western blot data using Image J software; (*bottom*) western blot images. **B**: Average relative expression of TGF-β1, PD-L1, and VEGF by normal kidney cell lines versus non-sarcomatoid ccRCC cell lines versus sarcomatoid ccRCC cell lines. **C1–3:** Percent expression of TGF-β1/β-actin, PD-L1/β-actin and VEGF/β-actin respectively for 43 non-sarcomatoid ccRCC patient tumor and adjacent normal tissue samples deciphered from the western blot data using Image J software. **C4–6:** Percent expression of TGF-β1/β-actin, PD-L1/β-actin and VEGF/β-actin respectively for 4 sarcomatoid ccRCC patient tumor and adjacent normal tissue samples deciphered from the western blot data using Image J software. Percent expressions of the three proteins of interest were independently calculated by dividing the value of the protein of interest (e.g., TGF-β1) of the tumor sample by the value of the housekeeping gene; this was also done for the normal tissue of the same patient; then, if the tumor sample value was higher both were divided by the tumor sample’s value which means the tumor sample would have 100 % expression whereas the normal sample’s value would be < 100 %. Whereas, if the normal sample’s value was higher, then both were divided by that value so that the normal tissue’s value would be 100 % and the tumor sample’s value would be < 100 %. The results are expressed as mean ± SD (*n* = 3). * *P* < 0.05, ** *P* < 0.01, *** *P* < 0.001, **** *P* < 0.0001, ns = not significant using paired *t*-test for C1–3 and Wilcoxon signed-rank test for C3–6. **D**: Percent expression of TGF-β1, PD-L1 and VEGF in non-sarcomatoid versus sarcomatoid tumor specimens. Percent expression was calculated as described above for panel C. **** *P* < 0.0001 using paired t-test.

Human ccRCC cell lines and ccRCC kidney tumor biopsies with adjacent normal kidney tissues were analyzed and characterized for expression of TGF-β1, PD-L1 and VEGF using western blotting. For the cell lines, two immortalized human renal proximal tubular epithelial cells derived from normal kidney cells (RPTEC and HK2), five non-sarcomatoid ccRCC cell lines (786-O, RCC4, RCC4/VHL, Caki-1 and RC2) and four ccRCC cell lines with sarcomatoid differentiation (RCJ41T1, RCJ41T2, RCJ41M and UOK127) were characterized and analyzed. As shown in (Fig. 1A), the normal cell lines (RPTEC and HK-2) expressed TGF-β1, PD-L1 and VEGF at relatively low levels, which was expected since TGF-β1, PD-L1 and VEGF are over-expressed in various types of cancer including ccRCC and expressed at low levels in normal tissues [29–31]. The average expression levels of TGF-β1, PD-L1 and VEGF were compared between the normal kidney cell lines and ccRCC cell lines. It was shown that the levels of the three targets were significantly higher in ccRCC cell lines regardless of sarcomatoid status, compared to normal kidney cell lines. Additionally, it was shown that the four cell lines with sarcomatoid features expressed higher levels of TGF-β1 and PD-L1 (*P* < 0.0001) and marginally lower levels of VEGF (*P* < 0.05) when compared to non-sarcomatoid cells (Fig. 1B). The forty-seven primary ccRCC tumors (obtained from previously untreated patients; forty-three being non-sarcomatoid and four being sarcomatoid) with matching adjacent normal tissues were analyzed to determine the relative expression levels of TGF-β1, PD-L1 and VEGF (Fig. 1C; western blots corresponding to Fig. 1C are provided in supplementary figure S1). The results indicated that in non-sarcomatoid ccRCC patient samples: TGF-β1 was significantly upregulated with 35/43 ccRCC tumors (81 %) demonstrating upregulation; PD-L1 was significantly upregulated with 28/43 tumors (65 %) demonstrating upregulation; while VEGF was upregulated in 26/43 tumors (60 %), however, this upregulation was not statistically significant. For sarcomatoid ccRCC patients, TGF-β1 was significantly upregulated with 4/4 tumors (100 %) demonstrating upregulation, whereas there was no statistically significant upregulation in PD-L1 expression (3/4 tumors (75 %)) and VEGF expression (2/4 tumors (50 %)). It was shown that relative levels of TGF-β1 and PD-L1 were significantly higher in sarcomatoid versus non-sarcomatoid ccRCC tumor biopsies; while VEGF levels were instead significantly lower (Fig. 1D). The clinical data on the primary RCCs were not available to the authors, however, based on the literature it is expected that the expression of TGF-β1, PD-L1 and VEGF is significantly higher in RCC tumors of high grades/stages than tumors with low grades/stages [32–35]. Collectively, the data in Fig. 1 indicate TGF-β1 and PD-L1 as candidate ‘druggable’ targets in ccRCC. Since the PD-1:PD-L1 axis already has FDA-approved drugs (e.g., nivolumab and avelumab) capable of impacting beneficially on survival in RCC patients, we chose to focus on targeting TGF-β1.

### 3.2. MSA specifically and directly binds to TGF-β1 which may cause growth inhibition in RCC

**Fig. 2.**
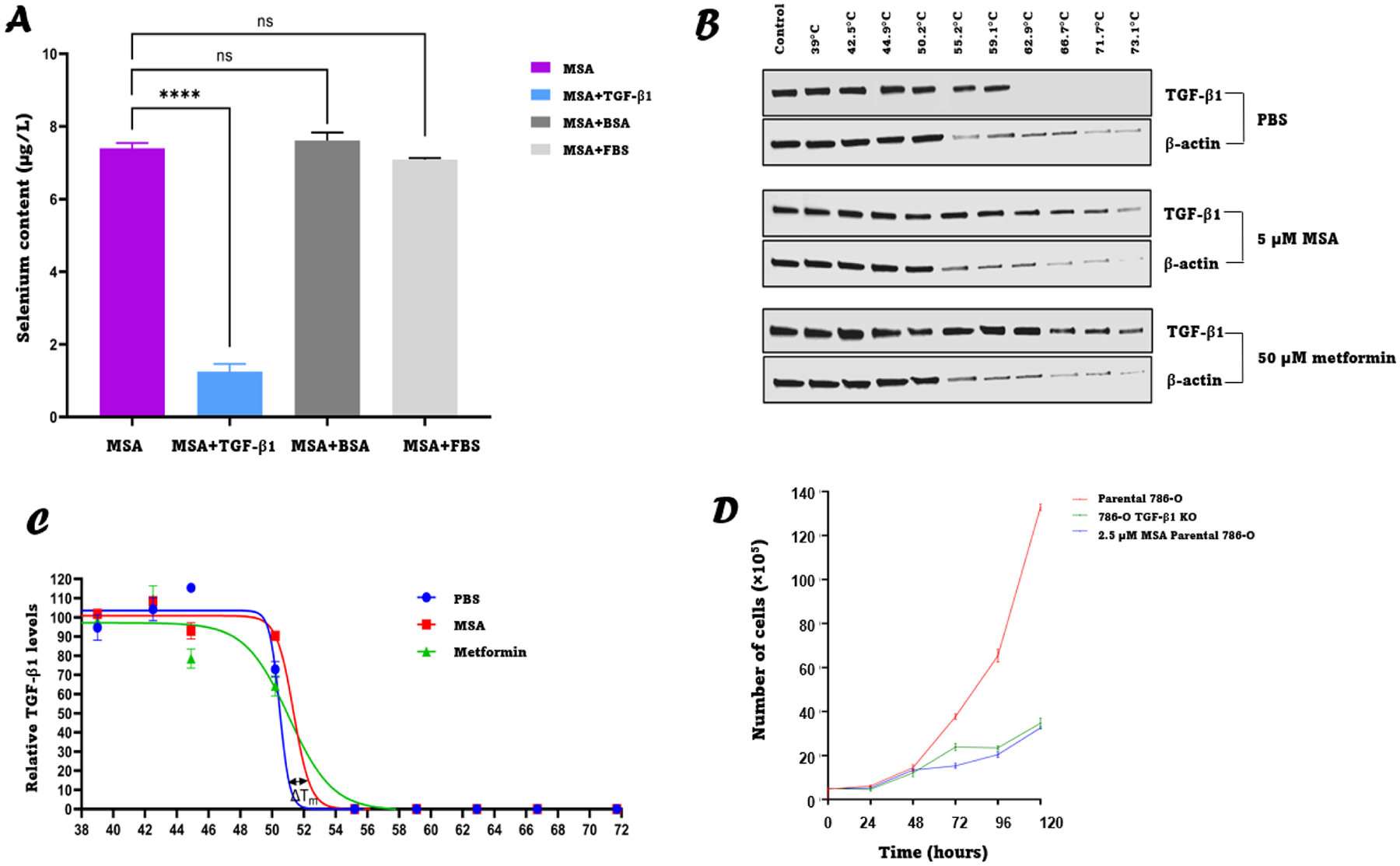
MSA binds directly and specifically to TGF-β1 and inhibits growth of 786-O cells. **A:** Selenium content (μg/l) in the filtrate of the MSA alone sample versus MSA plus the indicated proteins. **B:** Western blot images showing relative levels of TGF-β1 (in 786-O ccRCC cell line) after incubation with indicated treatment (PBS (negative control), 5 μM MSA (test) or 50 μM metformin (positive control)) at the indicated temperature. **C:** Thermostability of TGF-β1 as determined by the cellular thermal shift assay (as described in Materials and Methods section 2.5). **D:** In vitro growth curves of 786-O, 786-O TGF-β1 KO and 786-O treated with 2.5 μM MSA. Data represents the mean ± SD (*n* = 3). **** *P* < 0.0001, ns = not significant.

After establishing TGF-β1 to be an potentially important therapeutic target for ccRCC, we wished to test for a direct interaction between MSA and TGF-β1 using different approaches. The first approach involved mixing MSA with recombinant TGF-β1 at a 1:2 molar ratio, filtering this solution (using a 3 kDa pore filter to exclude TGF-β1 and selenium bound to TGF-β1) and then measuring selenium content in the filtrate and comparing to MSA alone samples. The results showed that selenium content in the filtrates from the MSA alone group was significantly higher than the MSA + TGF-β1 group, indicating that there was binding between MSA and TGF-β1 (Fig. 2A). To determine whether this binding is specific to TGF-β1 or whether MSA is binding non-specifically to proteins, two other general protein solutions, BSA and FBS, were tested as controls. The results showed that there was no significant difference between the selenium content of the filtrates from the MSA alone group compared to MSA with BSA or FBS groups, further indicating that the binding of MSA was specific to TGF-β1 (Fig. 2A). To further confirm that there was direct binding between MSA and TGF-β1, a cellular thermal shift assay was performed as described in the methods section. The results of this experiment showed that in the PBS treated cells (negative control) there were no detectable levels of TGF-β1 in the samples that were heated to temperatures ≥ 62.9 °C whereas there were detectable levels of TGF-β1 in both samples treated with either 5 μM MSA or 50 μM metformin (positive control) [36,37] at temperatures up to and including 73.1 °C (Fig. 2B). These results indicate that there was a shift in the melting temperature of TGF-β1 after binding to either MSA or metformin as shown in the curves in (Fig. 2C). One likely outcome of MSA binding to TGF-β1 is an effect on TGF-β1 function. TGF-β1 is known to promote growth of RCC although this can vary among cell lines. Therefore, we generated a 786-O cell line with TGF-β1 knocked out (786-O TGF-β1 KO), and compared in vitro growth over time of the 786-O TGF-β1 KO, with the parental 786-O, and the parental 786-O treated with 2.5 μM MSA. The in vitro growth curves showed that the growth of 786-O TGF-β1 KO is much slower compared to the parental 786-O and by treating parental 786-O with 2.5 μM MSA, the growth rate was similar to that of 786-O TGF-β1 KO (Fig. 2D). This experiment was repeated with 5 μM MSA and showed similar results (supplementary figure S6). In summary, the data provided by Figs. 2A–C indicate that MSA binds directly to TGF-β1 while Fig. 2D suggests that the binding of MSA to TGF-β1 has downstream antiproliferative consequences. However, the possibility that MSA specifically binds to other targets contributing to this effect cannot be ruled out.

### 3.3. TGF-β1, PD-L1, and VEGF are downregulated by MSA treatment in a dose-dependent manner

**Fig. 3.**
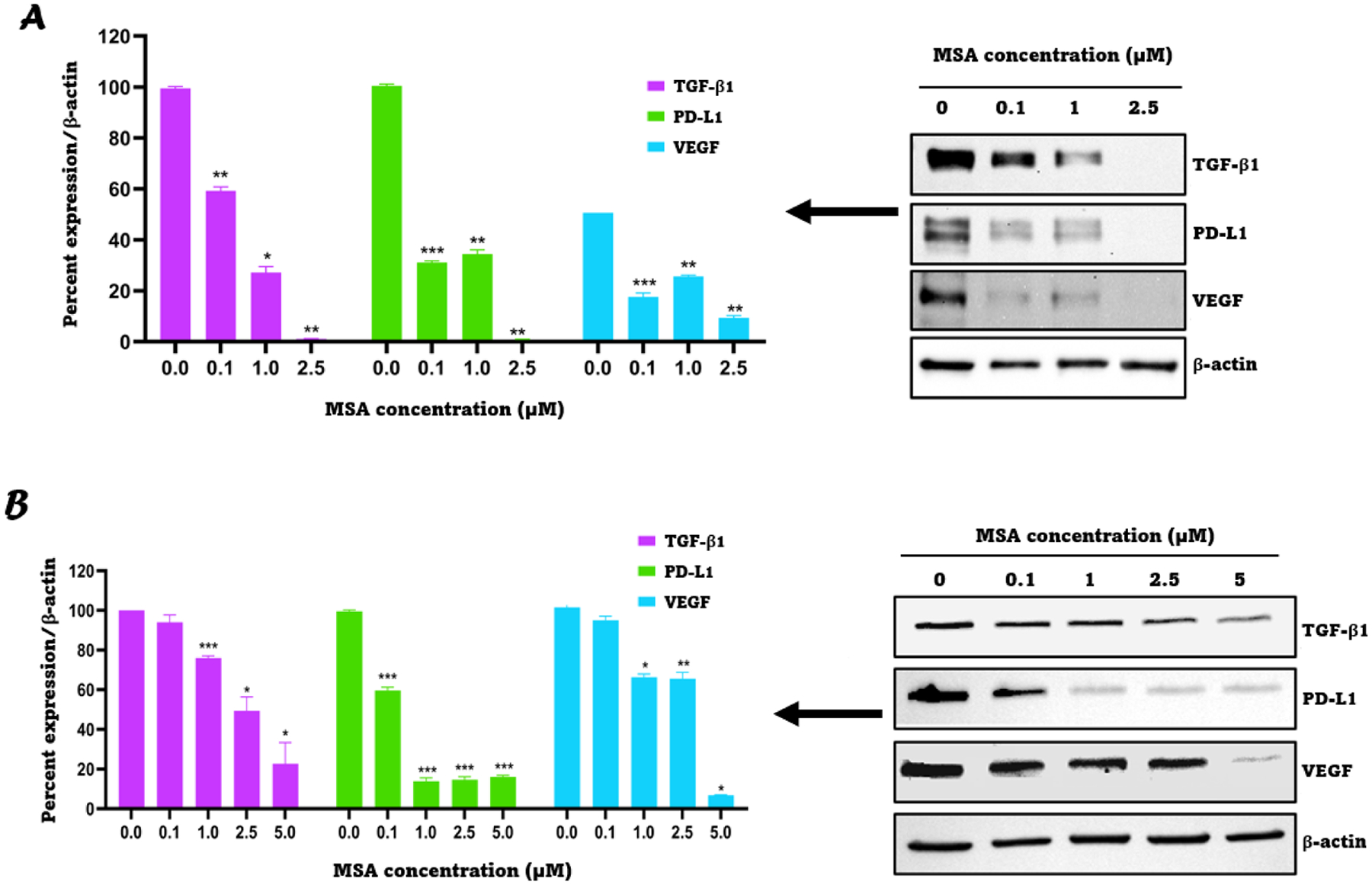
TGF-β1, PD-L1, and VEGF are downregulated by MSA in a dose-dependent manner. **A:** (left) Percent intensities deciphered from the western blot data using ImageJ software; (right) western blot images of TGF-β1, PD-L1, and VEGF for the RCJ41T1 cell line exposed to 0.1, 1 and 2.5 μM MSA for 24 h. **B:** (left) Percent intensities deciphered from the western blot using ImageJ software; (right) western blot images of TGF-β1, PD-L1, and VEGF for the 786-O cell line exposed to 0.1, 1, 2.5 and 5 μM MSA for 24 h. All results were expressed as the mean ± SD (*n* = 3). * *P* < 0.05, ** *P* < 0.01, *** *P* < 0.001.

After confirming the specific, and direct interaction between TGF-β1 and MSA, we investigated the effect of MSA treatment on the expression of TGF-β1, PD-L1 and VEGF by the non-sarcomatoid ccRCC cell line, 786-O, and the sarcomatoid ccRCC cell line, RCJ41T1; each chosen because they express high levels of TGF-β1, PD-L1 and VEGF. To study the dose-dependent effect of MSA exposure, the cells were treated with 0.1, 1 and 2.5 μM MSA for 24 h. Fig. 3 demonstrates that TGF-β1, PD-L1, and VEGF were significantly downregulated in a dose-dependent manner by MSA. Similar effects were also shown in two other cell lines (RCJ41T2 and RCJ41M) and the results are provided in supplementary figure S2.

### 3.4. TGF-β1, PD-L1 and VEGF are downregulated by MSA in a time-dependent manner

**Fig. 4.**
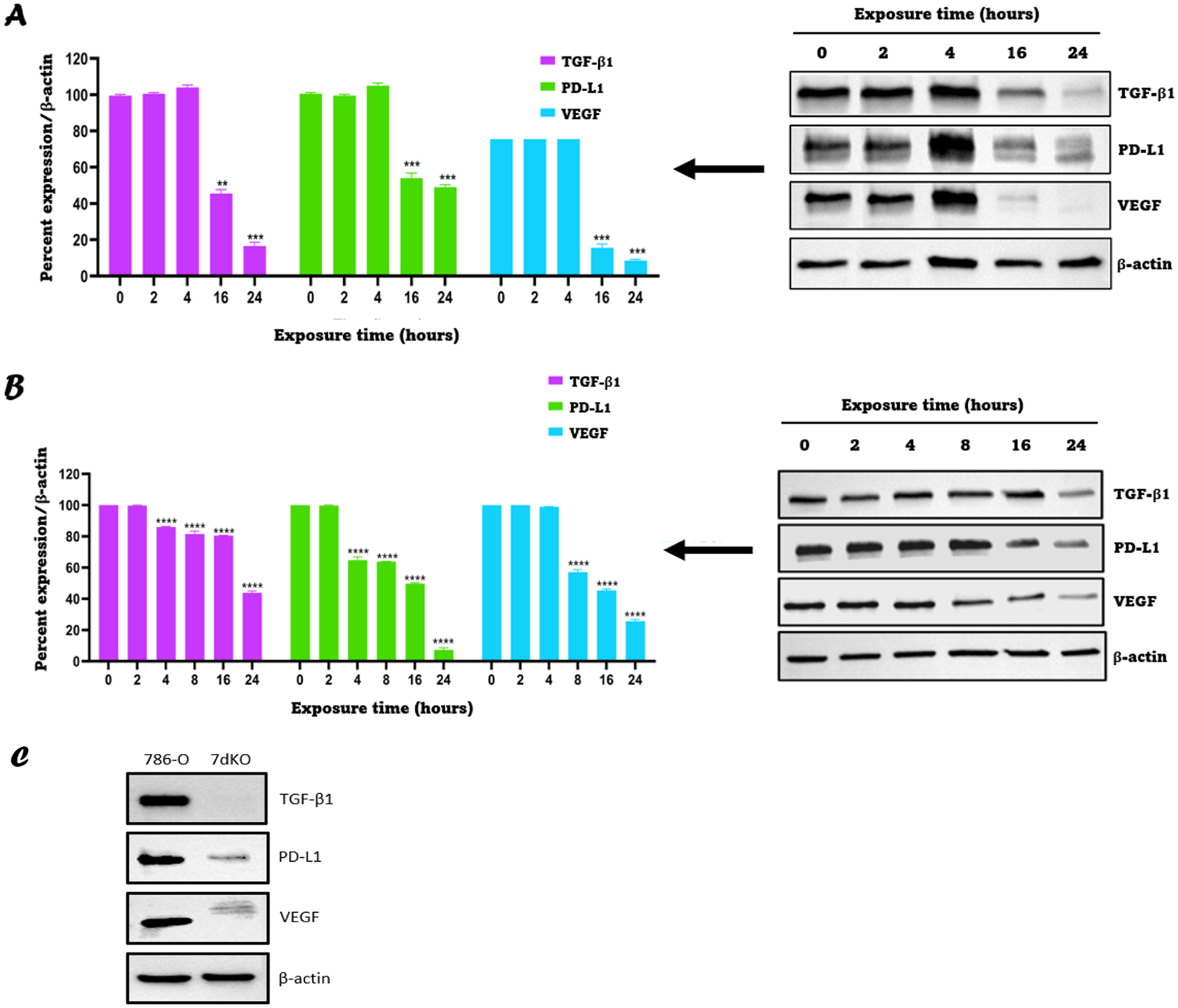
TGF-β1, PD-L1, and VEGF are downregulated in a time-dependent manner upon exposure to 2.5 μM MSA. **A:** (left) Percent intensities/relative expression of TGF-β1, PD-L1, and VEGF for RCJ41T1 cells exposed to 2.5 μM MSA for indicated time and deciphered from the western blot data using ImageJ software; (right) western blot images of TGF-β1, PD-L1, and VEGF for RCJ41T1 cells exposed to 2.5 μM MSA for indicated time. **B:** (left) Percent intensities/relative expression of TGF-β1, PD-L1, and VEGF for 786-O cells exposed to 2.5 μM MSA for indicated time and deciphered from the western blot data using ImageJ software; (right) western blot images of TGF-β1, PD-L1, and VEGF for 786-O cells exposed to 2.5 μM MSA for the indicated time. All results were expressed as the mean ± SD (*n* = 3). **C**: Western blotting results for TGF-β1, PD-L1 and VEGF from lysates obtained from parental 786-O cell line, 786-O TGF-β1 KO after 7 days selection. ** *P* < 0.01, *** *P* < 0.001, **** *P* < 0.0001.

Based on the results from the previous section, 2.5 μM MSA was chosen as the dose to investigate the time-dependent effect of MSA on the expression of TGF-β1, PD-L1 and VEGF. MSA was added to the RCJ41T1 and 786-O cell lines for 2, 4, 16, and 24 h. The results showed that TGF-β1, PD-L1, and VEGF expression were downregulated in a time-dependent fashion upon MSA exposure, with significant downregulation being observed for both cell lines at 16 h of exposure (Fig. 4). A similar effect was also observed with the RCJ41M cell line (supplementary figure S3). In order to determine whether it was more likely that the downregulation of PD-L1 and VEGF was due to the downstream consequences of TGF-β1 inactivation as opposed to direct action of MSA on each protein, we used the 786-O TGF-β1 KO cell line which was generated as described in the *methods*. Western blotting results comparing parental 786-O and 786-O TGF-β1 KO revealed that knocking out TGF-β1 resulted in concomitant downregulation of expression of PD-L1 and VEGF compared to the parental 786-O cell line (Fig. 4C). These findings indicate that TGF-β1 acts as an upstream regulator of PD-L1 and VEGF.

### 3.5. TGF-β1 expression recovers from the effect of 2.5 μM MSA in a time-dependent manner

**Fig. 5.**
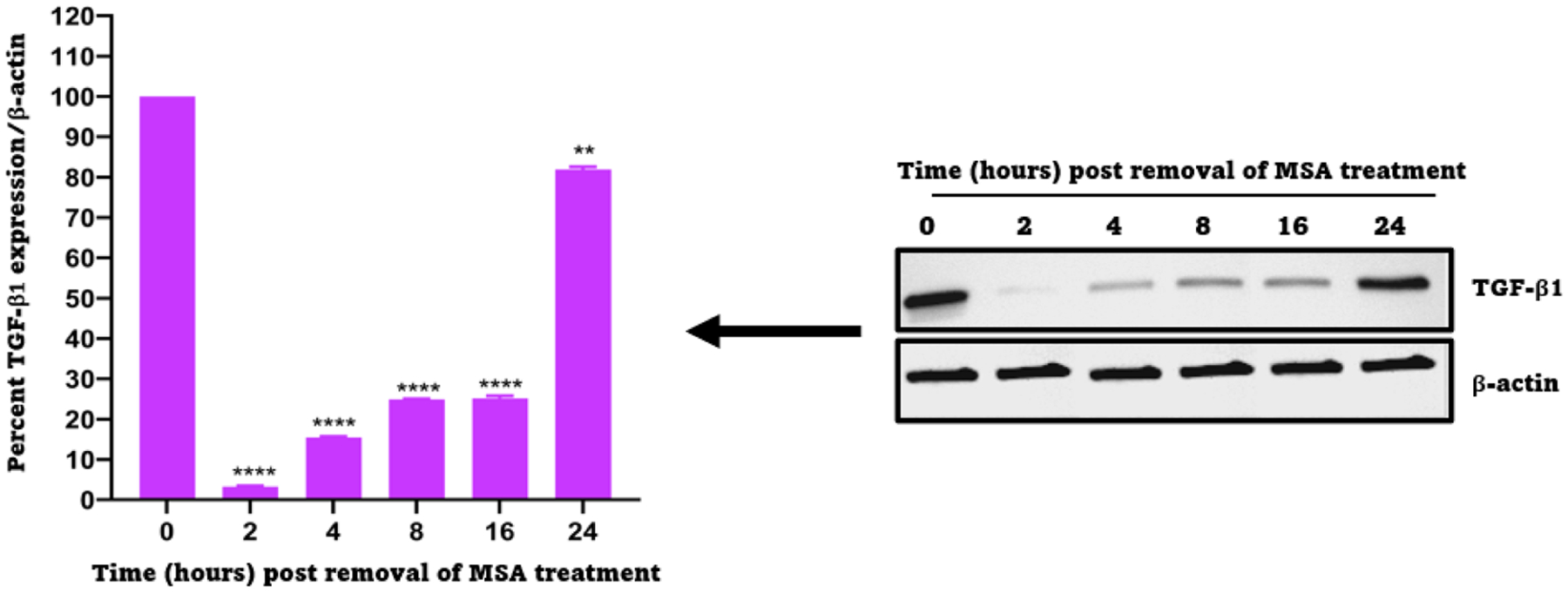
Recovery time of TGF-β1 expression from 2.5 μM MSA exposure in RCJ41T1 cells. (left) Percent intensities/relative expression deciphered from the western blot data using ImageJ software; (right) western blot images of TGF-β1 for RCJ41T1 cells after being exposed to 2.5 μM MSA for 24 h and monitoring the expression of TGF-β1 in MSA free media for 2, 4, 8, 16, and 24 h. The results were expressed as the mean ± SD (*n* = 3). ** *P* < 0.01, **** *P* < 0.0001.

After discovering that TGF-β1 is downregulated by MSA, it was crucial to know how long it takes for the expression of TGF-β1 to return to normal, as such information can determine dosing strategies in vivo. Thus, RCJ41T1 cells were treated with 2.5 μM MSA for 24 h, then the treatment was removed, and the cells were monitored over time for TGF-β1 expression using western blot analysis. It was revealed that TGF-β1 expression was approaching 80 % of its normal levels of expression within 24 h of the removal of the treatment (Fig. 5), suggesting that the daily use of selenium compounds in subsequent animal experiments may be a safe approach. We did not investigate the re-expression kinetics of TGF-β1 in the 786-O cell line, however, similar results were observed in all other cell lines tested (RCJ41M and RCJ41T2 cell lines) (supplementary figure S4).

### 3.6. SLM has anti-tumor activity in a nude mouse ccRCC xenograft model

**Fig. 6.**
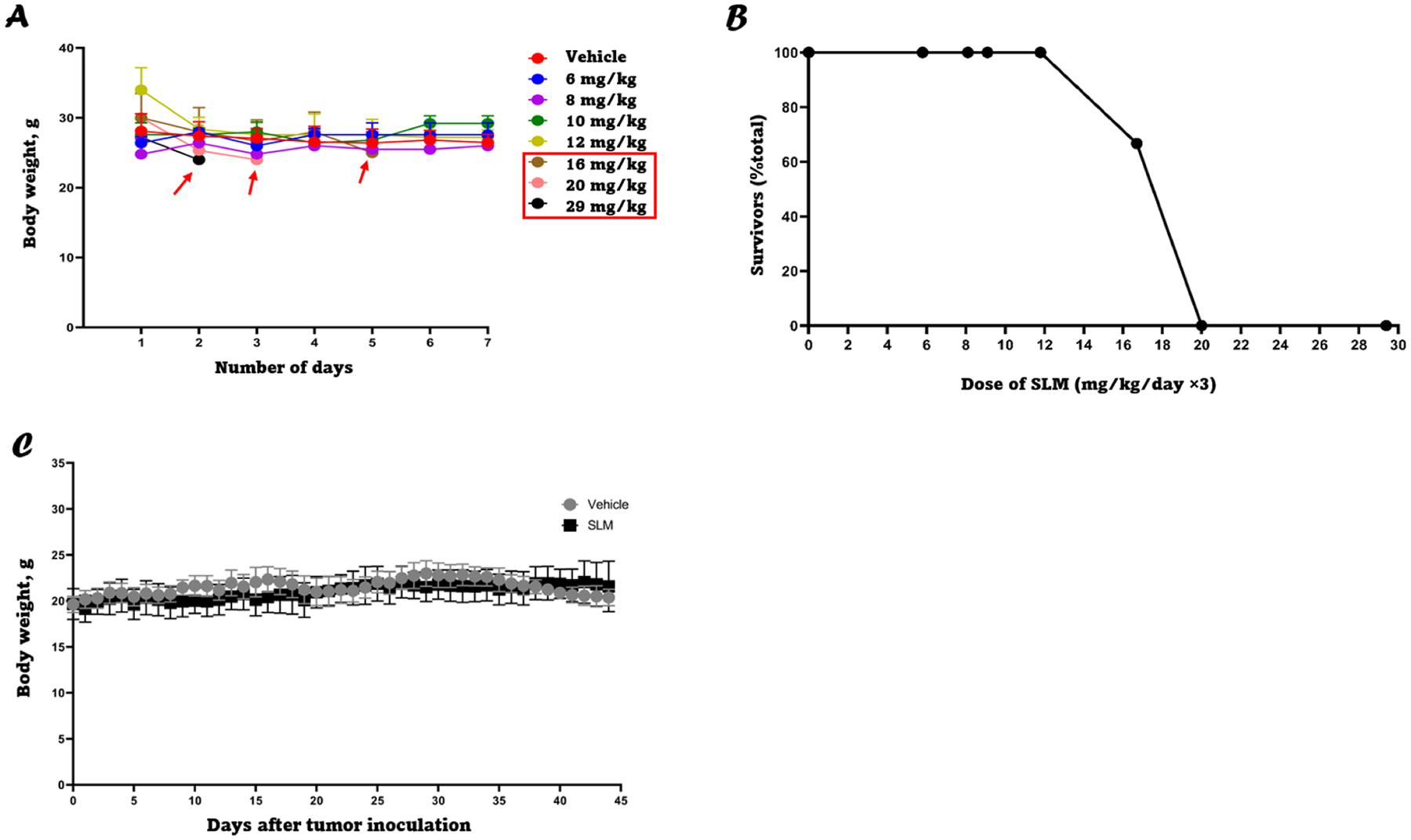
Toxicity study of SLM in athymic nude mice. **A:** Body weight of 6–8 weeks old/ athymic nude mice treated with various doses of SLM for three days and then monitored for up to 7 days to assess the acute toxicity of SLM. **B:** Dose-response curve of the lethal effect of a range of doses of SLM in athymic nude mice. **C:** Body weight of 6–8 weeks old athymic nude mice xenograft model treated with either a vehicle or 10 mg/kg SLM for 45 days.

**Fig. 7.**
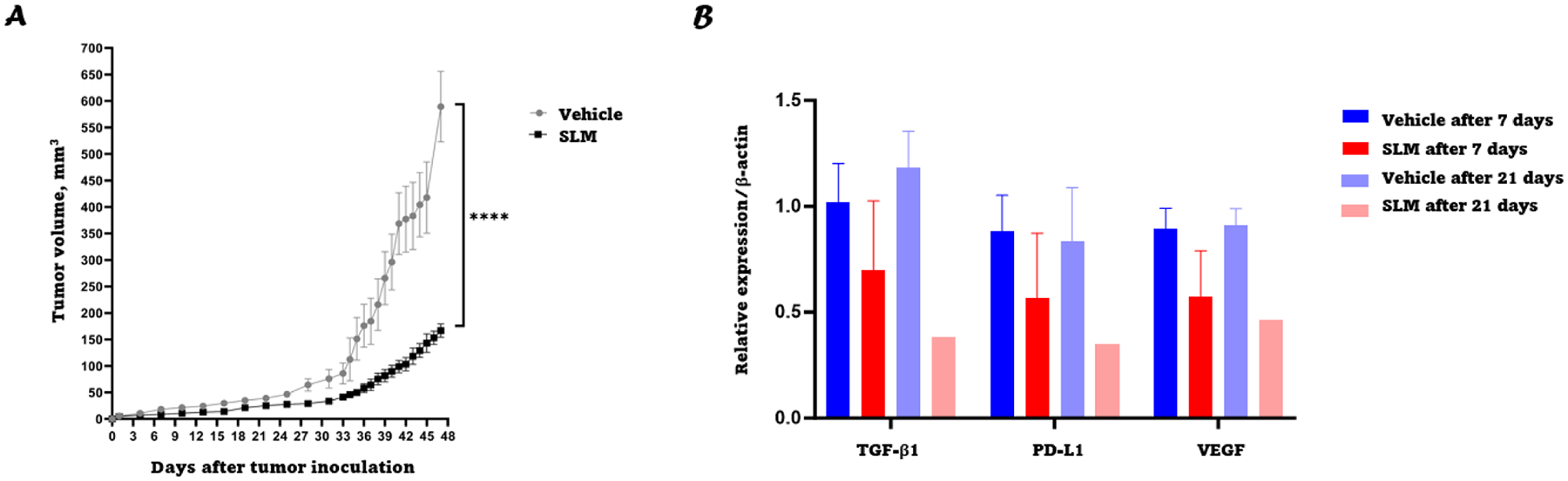
In vivo antitumor efficacy of SLM. **A:** 786-O tumor growth in athymic nu/nu mice treated with either deionized water or 10 mg/kg SLM. **B:** Relative expression of TGF-β1, PD-L1 and VEGF in mice tumor tissues after treatment with deionized water or SLM for 7 or 21 days as determined by western blotting. Data represents the mean ± SD. **** *P* < 0.0001.

After establishing that selenium (in the form of MSA) could downregulate TGF-β1 in vitro, selenium (in the form of SLM) was used to test if TGF-β1 can also be downregulated in vivo. The maximum tolerated dose of SLM was first assessed by administering increasing doses of SLM to female athymic nu/nu mice for 3 days and monitoring lethality and mouse body weight. SLM doses up to 14 mg/kg were considered safe and nontoxic with 100 % survival and without any significant weight loss. Doses higher than 14 mg/kg were toxic and lethal to some of the mice (Fig. 6A and B). Thus, the efficacy of 10 mg/kg SLM at abrogating tumor growth was evaluated by administering either deionized water or 10 mg/kg SLM to 786-O tumor-bearing female athymic nu/nu mice through oral gavage daily. Mice treated with SLM had average tumor volumes that were significantly smaller than those in the vehicle group (Fig. 7A; *P* < 0.0001). There was no difference in average body weight among the two groups, suggesting that the treatment was not toxic at the tested doses throughout the treatment period (Fig. 6C). Although not significant, there was a trend toward downregulation of TGF-β1 on days 7 and 21 in SLM treated mice compared to vehicle control (Fig. 7B), while PD-L1 and VEGF also followed a similar trend.

## 4. Discussion

Over recent years, treatments for ccRCC have improved significantly. The discovery of tyrosine kinase inhibitors (TKIs) and ICIs has revolutionized the way ccRCC is treated [7,9,38–40]. However, despite these successes, rapid resistance and dose-related toxicity often limit the long-term effectiveness of current therapies [41], and therefore ongoing research is required to unveil further improved therapeutic options [42].

There is an unmet need to identify additional ‘druggable’ targets altered in ccRCC tumors and to find an agent that may improve the efficacy of TKIs or ICIs and mitigate resistance to such therapies in ccRCC. TGF-β1 mRNA expression has recently been reported to be associated with poor prognosis in ccRCC [43], and as such may prove to be an important therapeutic target. TGF-β1 acts as a tumor suppressor in the early stages of ccRCC by preventing cell growth, initiating apoptosis, and inducing cell cycle arrest, however, TGF-β1 changes from being a tumor suppressor to a tumor promoter as ccRCC progresses [44,45]. Loss of the VHL gene, a common finding in ccRCC, interferes with TGF-β1 signaling consequently impairing its tumor-suppressive actions [46]. The dysregulation of TGF-β1 signaling stimulates EMT, angiogenesis, cancer invasion, and immune evasion [46]. Targeting the TGF-β1 pathway in ccRCC has emerged as a potential therapeutic strategy as it is considered one of the resistance mechanisms to various therapies in ccRCC [15]. Several approaches are being explored, including TGF-β1 receptor inhibitors, SMAD pathway inhibitors, and combination therapies targeting TGF-β1 along with other signaling pathways [47]. However, the main disadvantages of using these agents are their high toxicities as well as the high cost of treatment [48–51]. Thus, there is a need to find other options with higher safety profiles. A previous study using PC3, a human prostate cancer cell line, found that selenium inhibits the expression of TGF-β1 by TLR4-NF-κB signaling blockade [26]. Additionally, it has been noted in preclinical studies that selenium compounds, which are regarded to be less expensive than anti-TGF-β1 drugs, are safe and nontoxic when administered in dosages that show anti-tumor efficacy [52,53]. Thus, using selenium compounds to target TGF-β1 in ccRCC is a promising avenue of pursuit.

In our study, we initially investigated the potential of TGF-β1, as well as PD-L1 and VEGF, as therapeutic targets in ccRCC, by analyzing relative expression levels of these proteins in tumors from ccRCC patients as well as in a range of ccRCC cell lines. We found that TGF-β1 was overexpressed in ccRCC patients and ccRCC cell lines (Fig. 1), a finding that agrees with a previous report in a series of ccRCC cell lines (SKRC series) [46]. Relative expression levels of PD-L1 also indicated this protein to be a potential therapeutic target, however, since ICIs of PD-L1 are already approved for therapy in RCC and have shown therapeutic efficacy, we chose to focus on TGF-β1 instead. Using ccRCC cell lines, the interaction between TGF-β1 and MSA was investigated in 2 ways; by a simple mixing experiment, and a cellular thermal shift assay experiment. The results showed that there was a direct and specific interaction between TGF-β1 and MSA. These studies provide the first evidence of a direct and specific interaction between TGF-β1 and MSA. In vitro treatment of ccRCC cell lines with MSA showed downregulation of TGF-β1 in a dose- and time-dependent manner. The downregulation of TGF-β1 may increase the efficacy of TKIs and ICIs when used together in ccRCC treatments possibly by preventing the development of treatment resistance mechanisms facilitated by high levels of TGF-β1. Previous studies have shown that TGF-β1 downregulation can abrogate resistance through various mechanisms in different cancer types [54–56]. Our study here also showed that the recovery of TGF-β1 from MSA exposure is time-dependent and it is approaching 80 % from the baseline after 24 h suggesting that daily dosing of selenium in vivo would be an appropriate administration regimen.

The TGF-β1 KO 786-O cells generated in this research were used to explore whether the downregulation of PD-L1 and VEGF is due to the direct effect of MSA or is through TGF-β1 downregulation (Fig. 4c). The results indicated TGF-β1 to be an upstream regulator of PD-L1 and VEGF. Based on previous literature, this can be explained by the activation of prolyl hydroxylase domain enzyme upon TGF-β1 KO which resulted in HIF-1α degradation and the subsequent downregulation of VEGF and PD-L1 expression as shown in supplementary Figure S5 [57,58].

Our in vivo studies initially involved assessing the toxicity of increasing doses of SLM in athymic nude mice in terms of body weight reduction and lethality. A dose of 10 mg/kg was determined to be safe which has been previously reported [53]. Nude mice were challenged with 786-O cells and were subsequently treated with either vehicle or 10 mg/kg SLM. The in vivo study revealed that 10 mg/kg has an anti-tumor activity. Given the previous in vitro findings, this may be at least partially attributed to downregulation of TGF-β1 expression. Our group is the first to recognize that TGF-β1, which is known to be overexpressed in ccRCC and other types of cancer, can be downregulated by MSA. In addition to TGF-β1 downregulation by MSA, it was also shown that MSA could promote the downregulation of PD-L1 and VEGF and is supported by a study carried out by Wang et al. demonstrating that TGF-β1 inhibits the expression of VEGF-A in colon cancer [59]. Meanwhile, several studies have shown that TGF-β1 can promote tumor progression, invasion, and aggressiveness [60] of various types of tumors particularly during the late stages of the disease. It is likely that reduction in the expression levels of PD-L1 and VEGF will result in enhanced responses to the standard doses of inhibitors. Finding a treatment-modulating agent like selenium that is cheap, safe, and non-toxic and utilizing it in combination with anti-angiogenic agents and/or immunotherapies for the treatment of ccRCC should prove to be a boon for ccRCC patients with advanced disease. Finally, it is possible that aside from affecting TGF-β1 expression and function, selenium compounds may induce a range of responses in ccRCC cell lines. Whilst our study focused specifically on examining how selenium affects TGF-β1 expression to evaluate its anti-tumor properties, future research should consider conducting RNAseq on RCC cell lines treated with selenium and comparing the resultant data with untreated RCC cell lines to better understand the extent of selenium’s pleiotropic effects. It would also be valuable to examine TGF-β1 mRNA levels after selenium treatment to corroborate our results as we would expect to see a reduction in TGF-β1 mRNA levels following selenium treatment considering that TGF-β1 is capable of regulating its own expression [61]. Additionally, the cytotoxic effect of MSA was not investigated in our research, but it was reported in the literature that there are many mechanisms by which MSA causes cytotoxicity including reactive oxygen species (ROS) generation, lipid peroxidation, and induction of apoptosis [21,62,63].

## 5. Conclusions

TGF-β1 is a potential therapeutic target in ccRCC and other types of cancer. It was shown here to be significantly more highly expressed in patient tumor tissue samples compared to normal adjacent kidney samples and in sarcomatoid ccRCC compared to non-sarcomatoid ccRCC. TGF-β1 is downregulated in vitro by MSA in a dose- and time-dependent manner and this downregulation appeared to be the result of a direct, specific binding between TGF-β1 and MSA. Additionally, 10 mg/kg SLM demonstrated antitumor activity in 786-O tumor-bearing athymic nude mice and this antitumor activity may be at least partially attributed to the downregulation of TGF-β1 expression in the tumor tissues.

Overall, these findings are of a high importance, and if we can demonstrate that downregulation of TGF-β1, VEGF and PD-L1 in vivo can produce a long-lasting response and mitigate resistance when using effective, non-toxic doses of selenium compounds in combination with other ccRCC treatment options, this would promote the initiation of a new clinical trial in patients with advanced ccRCC. If successful, this new and novel approach could lead to more than incremental improvements in ccRCC treatment.

## Supplementary Material

1

## Data Availability

The data generated in this study are available upon request from the corresponding author.
