# Supplementary material for: Targeting transforming growth factor-β1 by methylseleninic acid/seleno-L-methionine in clear cell renal cell carcinoma: Mechanisms and therapeutic potential
Source: Cancer Treat Res Commun. Author manuscript; Available in PMC 2025 Feb 22. (PMC11846624; doi:10.1016/j.ctarc.2025.100864)
Supplement: 1 [file NIHMS2056135-supplement-1.docx]

**Supplementary figures:**


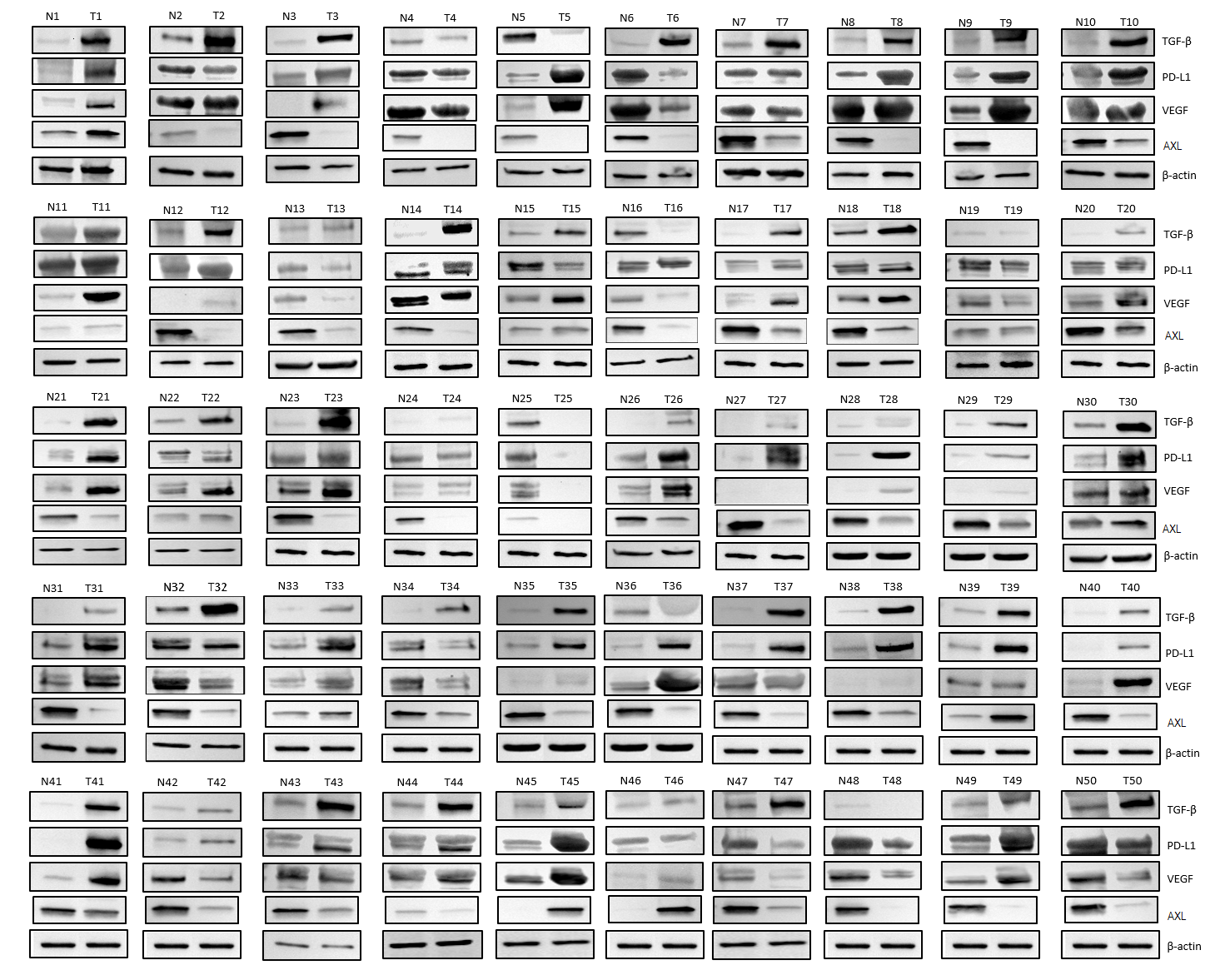


**Supplementary Figure S1:** Western blots for the expression of TGF-β, PD-L1, and VEGF in the 50 ccRCC patients. Each block represents one patient with N means adjacent normal tissue and T means tumor tissue. For example, N1 is the adjacent normal tissue for patient 1 and T1 is the corresponding tumor tissue for the same patient.


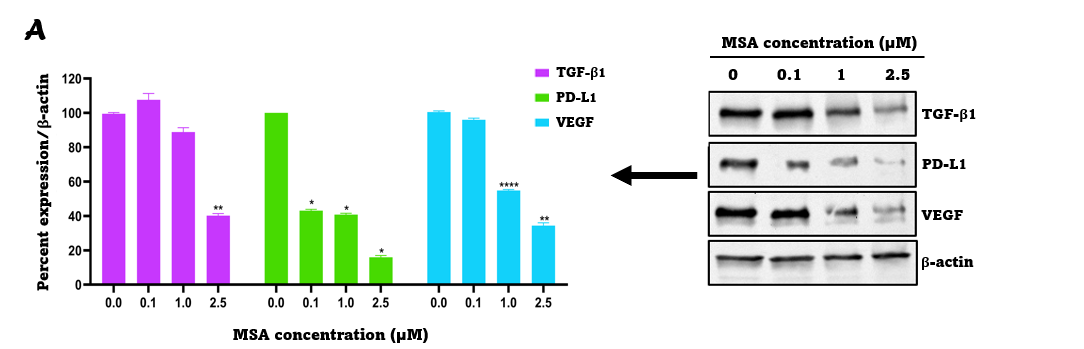


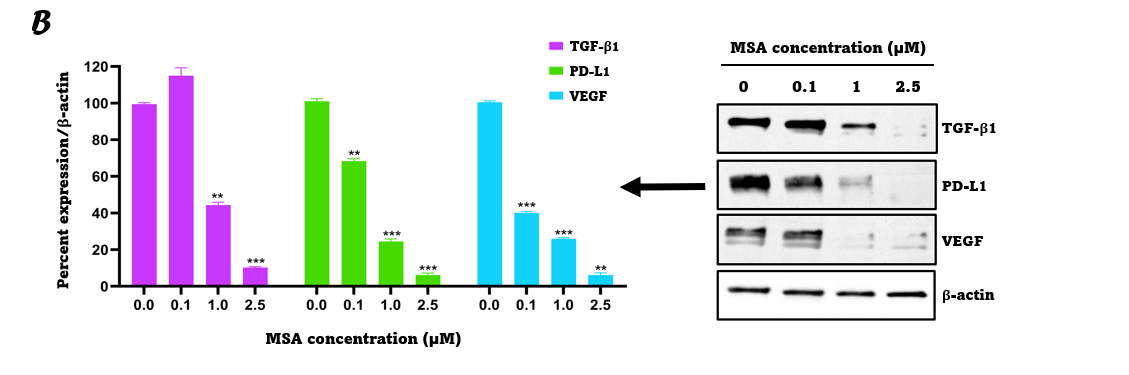


**Supplementary Figure S2:** TGF-β1, PD-L1, and VEGF are downregulated by MSA in a dose-dependent manner. **A:** (left) shows the percent intensities deciphered from the western blot using ImageJ software; (right)western blot images of TGF-β1, PD-L1, and VEGF for RCJ41Mcells exposed to 0.1, 1 and 2.5 μM MSA for 24 hours. **B:** (left) shows the percent intensities deciphered from the western blot using ImageJ software; (right) western blot images of TGF-β1, PD-L1, and VEGF for RCJ41T2 cells exposed to 0.1, 1 and 2.5 μM MSA for 24 hours. All results were expressed as the mean ± SD (n=3). * P <0.05, ** P <0.01, *** P <0.001, **** P <0.0001.


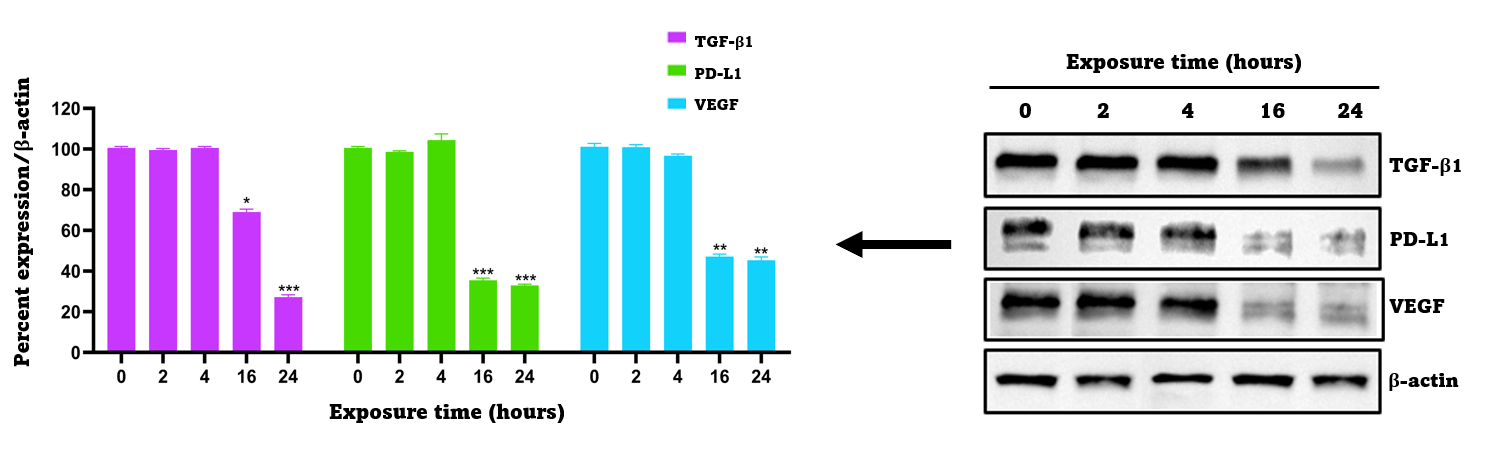


**Supplementary Figure S3:** TGF-β1, PD-L1, and VEGF are downregulated in a time-dependent manner upon exposure to 2.5 μM MSA. (left) shows the percent intensities/relative expression of TGF-β1, PD-L1 and VEGF for RCJ41M cells exposed to 2.5 µM MSA for indicated time and deciphered from the western blot using ImageJ software; (right) western blot images of TGF-β1, PD-L1, and VEGF for RCJ41M cells exposed to 2.5 µM MSA for indicated time. All results were expressed as the mean ± SD (n=3). * P <0.05 ** P <0.01, *** P <0.001.


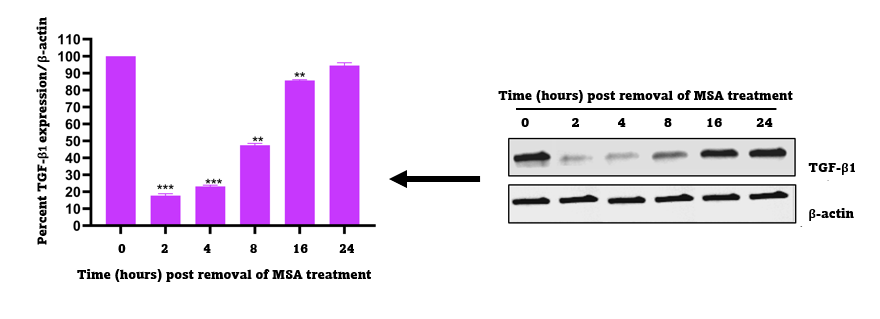


B

A


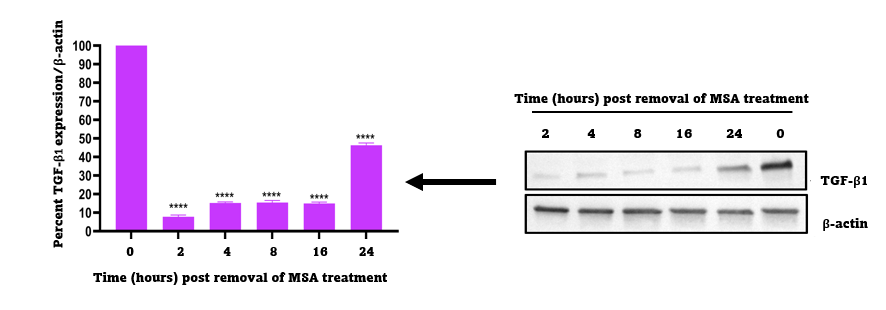


**Supplementary Figure S4:** Recovery time of TGF-β1 expression from 2.5 μM MSA exposure in RCJ41T2 and RCJ41M cells. **A:** (left) shows the percent intensities deciphered from the western blot using ImageJ software; (right) Western blot images of TGF-β1 for RCJ41T2 cells after being exposed to 2.5 μM MSA for 24 hours and monitoring the expression of TGF-β1 in MSA free media for 2, 4, 8, 16, and 24 hours. **B:** (left) shows the percent intensities deciphered from the western blot using ImageJ software; (right) Western blot images of TGF-β1 for RCJ41M cells after being exposed to 2.5 μM MSA for 24 hours and monitoring the expression of TGF-β1 in MSA free media for 2, 4, 8, 16, and 24 hours. All results were expressed as the mean ± SD (n=3). ** P <0.01, *** P <0.001, **** P <0.0001.


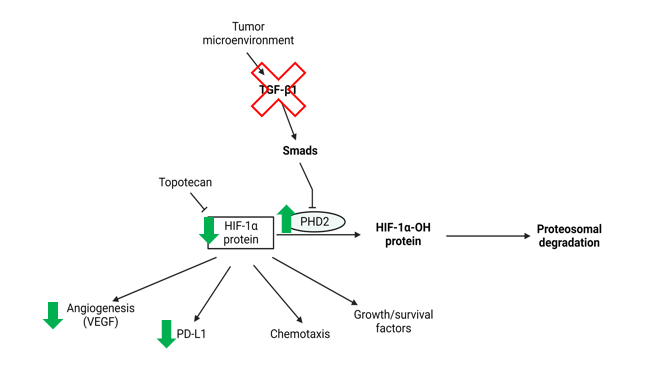


**Supplementary Figure S5:** TGF-β1 and HIF-1α pathway and their downstream effect on angiogenesis, chemotaxis, growth, and survival


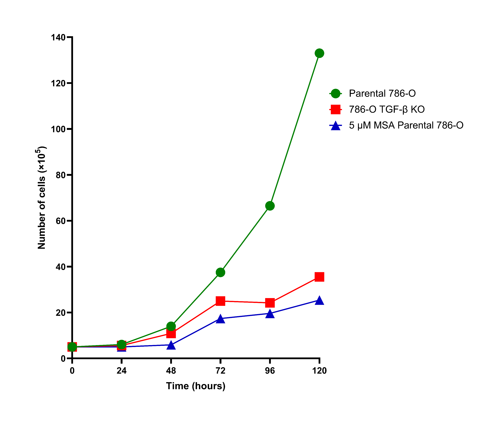


**Supplementary Figure S6:** In vitro growth curves of 786-O, 786-O TGF-β1 KO and 786-O treated with 5 µM MSA.


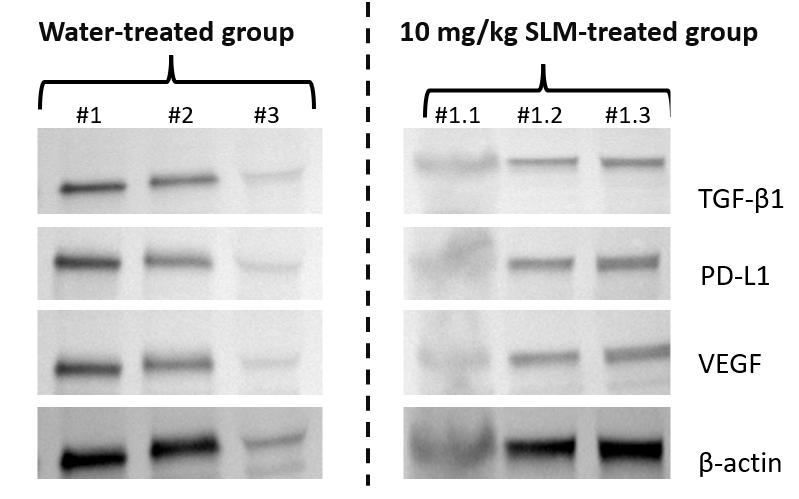

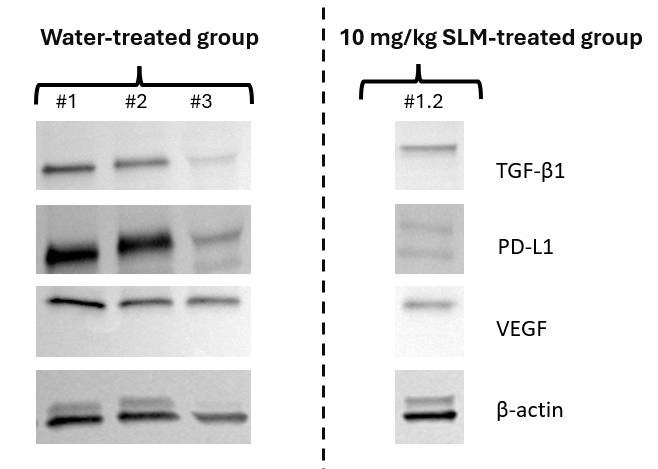


A

B

**Supplementary Figure S7:** Western blots for nude mice xenograft tissues collected at **A:** Day 7. **B:** Day 21.


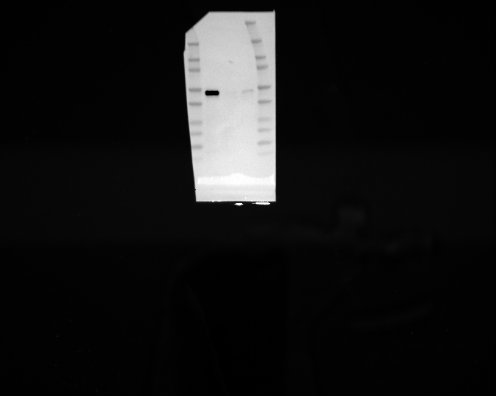

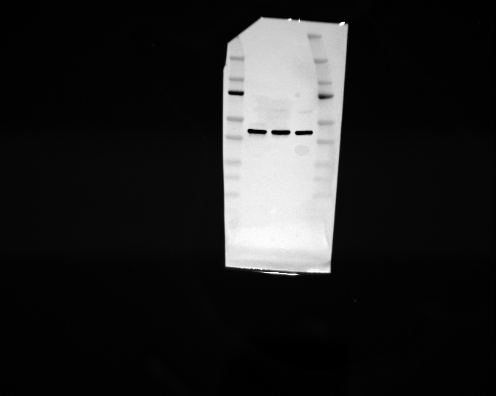


**Supplementary Figure S8:** Uncut Western blots for TGF-β1 expression for control 786-O cells, 786-O KO for TGF-β1 after 7 days of selection, and 786-O KO for TGF-β1 after 3 days of selection. The figure on the left is the TGF-β1 expression and the one on the right represents the β-actin expression of the same blot after stripping the membrane with a stripping buffer.


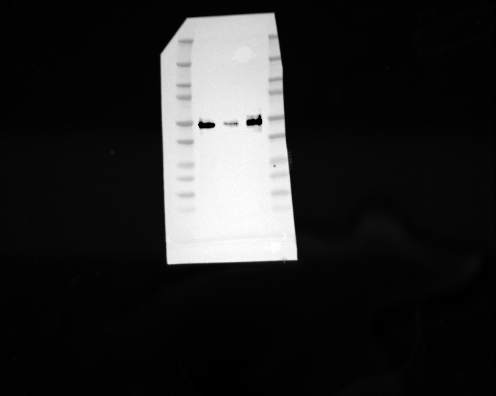

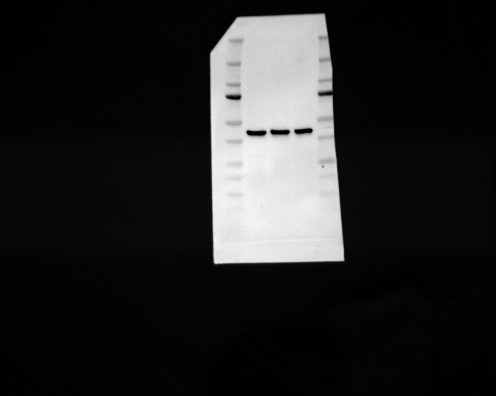


**Supplementary Figure S9:** Uncut Western blots for PD-L1 expression for control 786-O cells, 786-O KO for TGF-β1 after 7 days of selection, and 786-O KO for TGF-β1 after 3 days of selection. The figure on the left is the PD-L1 expression and the one on the right represents the β-actin expression of the same blot after stripping the membrane with a stripping buffer.


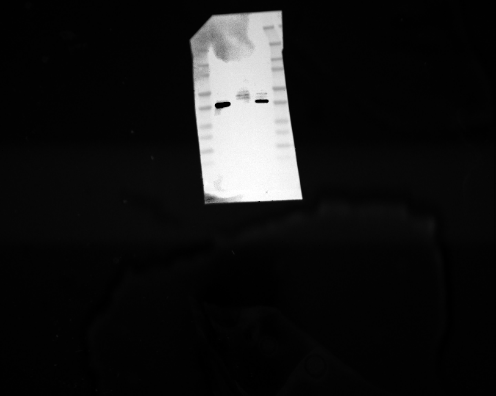

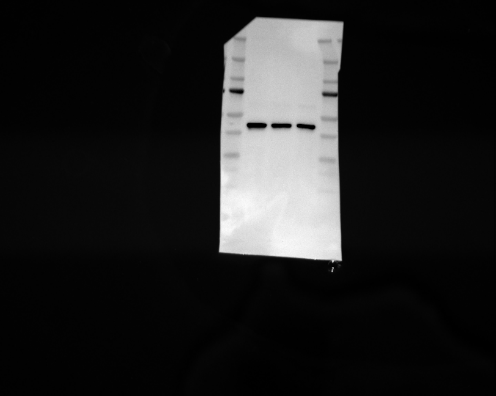


**Supplementary Figure S10:** Uncut Western blots for VEGF expression for control 786-O cells, 786-O KO for TGF-β1 after 7 days of selection, and 786-O KO for TGF-β1 after 3 days of selection. The figure on the left is the VEGF expression and the one on the right represents the β-actin expression of the same blot after stripping the membrane with a stripping buffer.
